# Cognitive function and risperidone long-acting injection vs. paliperidone palmitate in schizophrenia: a 6-month, open-label, randomized, pilot trial

**DOI:** 10.1186/s12888-016-0883-9

**Published:** 2016-05-29

**Authors:** Yoshiteru Takekita, Yosuke Koshikawa, Chiara Fabbri, Shiho Sakai, Naotaka Sunada, Ai Onohara, Keiichiro Nishida, Masafumi Yoshimura, Masaki Kato, Alessandro Serretti, Toshihiko Kinoshita

**Affiliations:** Department of Biomedical and NeuroMotor Sciences, University of Bologna, Viale Carlo Pepoli 5, Bologna, 40123 Italy; Department of Neuropsychiatry, Kansai Medical University, 10-15 fumizono-cho, Moriguchi-shi, Osaka 570-8507 Japan

**Keywords:** Schizophrenia, Risperidone long-acting injection, Paliperidone palmitate, Cognitive function, Efficacy, Subjective well-being

## Abstract

**Background:**

Recently, long-acting injection (LAI) of second-generation antipsychotics has become a valuable strategy for the treatment of schizophrenia. However, few studies have compared the effects of different LAI antipsychotics on cognitive functions so far. The present study aimed to compare the influence of risperidone LAIs (RLAI) and paliperidone palmitate LAIs (PP) on cognitive function in outpatients with schizophrenia.

**Methods:**

In this 6-month, open-label, randomized, and controlled study, 30 patients with schizophrenia who were treated with RLAIs were randomly allocated to the RLAI-continued group or the PP group. At baseline and 6 months, the patients were evaluated using the Brief Assessment of Cognition in Schizophrenia (BACS) that was the primary outcome of the study. The Subjective Well-being under Neuroleptic drug treatment-Short form (SWNS), the Positive and Negative Syndrome Scale (PANSS), and the Drug-Induced Extrapyramidal Symptoms Scale (DIEPSS) scores were secondary outcome variables and they were tested at the same time points.

**Results:**

The two groups did not differ in terms of PANSS, DIEPSS, or SWNS total score changes. However, the BACS score for the attention and processing speed item showed higher improvement in the PP group than the RLAI group (*p* = 0.039).

**Conclusions:**

The results of this preliminary study suggest that PPs may improve attention and processing speed more than RLAIs. Anyway, a replication in a larger and double-blind study is needed.

**Trial registration:**

UMIN000014470. Registered 10 July 2014

**Electronic supplementary material:**

The online version of this article (doi:10.1186/s12888-016-0883-9) contains supplementary material, which is available to authorized users.

## Background

Schizophrenia is a devastating disease that has a chronic or intermittent course with numerous relapses over time [1]. Relapses of schizophrenia are known to adversely affect many biological functions [2] and antipsychotic treatment is pivotal for preventing relapses [3]. Anyway, in clinical setting patients’ compliance to oral antipsychotics is often poor and difficult to maintain over time [4], resulting in a heavy impact on the risk of relapse [5].

Previous studies reported that several factors are related to the adherence to antipsychotic drugs, many of them are patient-related factors (e.g. symptom severity, illness insight, attitude to medication or subjective well-being), but there are also medication-related factors (e.g. side effects), and environment-related factors (e.g. therapeutic alliance) [6–9]. One possible approach to manage patient non-adherence is the use of long-acting injections (LAI) [10]. Many studies have reported remarkable improvements in psychiatric symptoms and/or a higher efficacy in preventing recurrence/relapse when patients with schizophrenia are treated with LAIs [11].

Among the second-generation antipsychotics (SGAs), the present study examined risperidone (RIS) LAIs (RLAI) and paliperidone (PAL) palmitate LAIs (PP) that was recently marketed in many countries. PP is an active metabolite of RIS, and they resemble each other in many aspects. However, they differ in some of their pharmacological features [12]. Indeed, previous clinical studies compared Oral-RIS and Oral-PAL and they reported some differences pertaining the effects on cognitive functions [13, 14]. The treatment of neurocognitive deficits is another key target of treatment [15], since deficits in neurocognitive functions are among the core symptoms of schizophrenia [16]. Although some trials investigating cognitive functions in patients treated with RLAI were performed, PP was never investigated in relation to that issue [17, 18]. No study directly comparing RLAI and PP was performed so far, neither comparing their effects on cognitive functions.

Accordingly, the present paper aims to compare the influence of RLAIs and PPs on cognitive functions using the Brief Assessment of Cognition in Schizophrenia (BACS) score as the primary outcome. As secondary outcomes, we investigated the differences between the drugs on measures of psychiatric symptoms, extrapyramidal symptoms, and subjective well-being, which are relevant issues in regard to long-term LAI use and adherence to antipsychotics.

## Methods

### Study design and setting

**Fig. 1:**
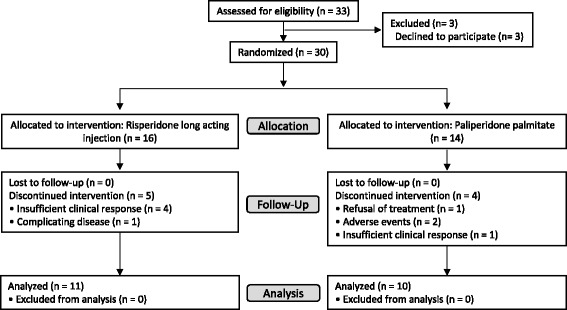
CONSORT flow of participants through the trial

This was a 24-week pilot study using an open-label, parallel, randomized controlled design. It was conducted at Kansai Medical University Takii Hospital in Osaka from July 2014 to February 2015, in accordance with the Declaration of Helsinki. The trial protocol was approved by the institutional review board at Kansai Medical University. After a full description of the study, all participants provided written informed consent prior to entering the study. We described this clinical trial according to the CONSORT 2010 guidelines. Enrollment, allocation, and follow-up of the study patients are depicted in the CONSORT diagram (Fig. 1). A supporting CONSORT 2010 checklist is available as supplementary information (see Additional file 1).

### Patients

All patients were at least 20 years old and had been diagnosed with schizophrenia or schizoaffective disorder based on the DSM-IV-TR criteria. The inclusion criteria were: 1) being during a non-acute phase of disease; 2) Positive and Negative Syndrome Scale (PANSS) total score of 120 or less; and 3) having received RLAI for 2 months or longer. The exclusion criteria were; 1) comorbid serious physical disorder; 2) active suicidal ideation; 3) history of attempted suicide; 4) history of drug or alcohol abuse; 5) mental retardation; 6) pregnancy; 7) current treatment with Oral-RIS, Oral-PAL; or 8) current treatment with multiple oral antipsychotics. Intelligence quotient was assessed using the Japanese Adult Reading Test (JART) [19]. For the duration of the study, subjects were allowed to take anticholinergic agents for new extrapyramidal symptoms, drugs for concomitant medical conditions if they started taking them before the enrollment in the study, low-dose sleep-inducing medications as needed, and other drugs considered to have no effect on the outcomes of interest. Additional administration of new antipsychotics, except for RLAI and PP, was not allowed during the study.

### Procedures

Patients satisfying the inclusion criteria were randomized 1:1 to either the RLAI-continued group (hereafter, the RLAI group) or the PP group using the random number generation program of the SPSS software, version 21 (IBM SPSS, Tokyo, Japan). Patients in the RLAI group continued their treatment with the same dose of RLAI, and then underwent intramuscular injection into the gluteal muscles every 2 weeks. The dose was determined depending on each patient’s clinical status, with an upper limit of 50 mg/2 weeks. Patients in the PP group started the treatment by intramuscular injection into the deltoid or gluteal muscles. Their starting dose was equivalent to twice the dose of RLAI [20]. In the present study, a 4-week PP preparation was used. The dose was determined depending on each patient’s clinical status, with an upper limit of 150 mg/4 weeks.

### Outcome measures

The primary outcome was the baseline-6 month change in BACS score between the RLAI group and the PP group. The secondary outcomes were the baseline-6 month changes in subjective well-being, psychiatric symptoms, and extrapyramidal symptoms between the two groups. Finally, the correlation between the changes in cognitive function and the changes in subjective well-being were evaluated.

The following assessments were performed at baseline and after 24 weeks: the BACS, Japanese version for cognitive function [16]; the Subjective Well-being under Neuroleptic drug treatment Short form (SWNS) [21, 22], Japanese version for subjective well-being; PANSS for clinical psychopathology [23]; and the Drug-Induced Extrapyramidal Symptoms Scale (DIEPSS) for extrapyramidal symptoms [24].

The BACS is a tool for measuring cognitive function in patients with schizophrenia that consists of six items: verbal memory, working memory, motor speed, verbal fluency, attention, processing speed, and executive function [25]. Sufficient reliability and validity have been established for the BACS Japanese version [16]. In accordance with several previous studies using the BACS, the primary outcome measures were standardized into z-scores (the mean of healthy controls was set to zero, and the standard deviation was set to one) [25–27]. In the present study, the data for healthy controls were obtained from a previous study [28]. The BACS score was adjusted for age using age-matched controls to calculate the BACS-J z-scores for each schizophrenia patient in the present study.

The SWNS is a tool for measuring subjective well-being in patients with schizophrenia undergoing antipsychotic treatment. It consists of 20 questions covering five categories: mental function, self-control, emotional regulation, physical functioning, and social integration. Sufficient reliability and validity have been established for the SWNS Japanese version [29]. Each question is answered using a 6-point scale, with higher scores indicating higher subjective well-being.

Due to the open-label design of the study, neither patients nor raters were blind to patients’ group assignment.

### Statistical analysis

The analysis was performed only for patients who completed all the 24 weeks of the study. The raw data collected at baseline and endpoint were used for the analysis. To ensure group comparability, baseline clinical characteristics were tested by t-tests or Pearson’s chi-square tests as appropriate. Analysis of covariance (ANCOVA) was used to assess the change of BACS z-scores, SWNS scores, PANSS scores, DIEPSS, and antipsychotic dose in each treatment group using the baseline score as covariate. Only in the analysis of BACS z-score change the antipsychotic dose was used as covariate in addition to the baseline score because a significant correlation was observed between the BACS z-score and the antipsychotic dose. An exploratory Pearson’s correlational analysis was used to determine potential associations between changes in cognitive and subjective well-being scores. Analyses were performed with SPSS software, version 21.0 J (SPSS, Tokyo, Japan). All statistical tests were two-tailed, and a *p*-value less than 0.05 was considered significant.

## Results

### Demographic and clinical characteristics

Thirty patients were allocated into the two groups (RLAI group, *n* = 16; PP group, *n* = 14) at the start of the study. During the study, 5 patients from the RLAI group and 4 from the PP group dropped out of the study (Fig. 1). Thus, the final analysis included the 21 patients who completed the study (11 in the RLAI group and 10 in the PP group). The clinical-demographic characteristics of the sample are shown in Table 1. At baseline, the two groups did not differ significantly in age, onset, sex, diagnosis, PANSS total score, DIEPSS total score (sum of scores for items 1 to 8), total antipsychotics dose (chlorpromazine equivalent), or concomitant medications. Concomitant medications that a patient have started before the inclusion in the study were allowed to continue until the end of the study. During the 24-week study period, two patients from the PP group were also treated with low-dose ultrashort-acting hypnotics for new insomnia, and one patient from the same group was treated with an anti-hyperlipidemia drug for new hyperlipidemia. The patients were not treated with other drugs, including psychotropics.

**Table 1:** Demographic and Clinical Characteristics of Patients

	RLAI group	PP group	*p*
Age (years)	46.4 ± 10.4	43.5 ± 11.8	NS
Onset (years)	29.5 ± 11.9	32.9 ± 11.7	NS
Sex (Male/Female)	7/4	4/6	NS
Diagnosis (schizophrenia/schizoaffective disorder)	11/0	9/1	NS
PANSS total score	83.0 ± 19.9	78.1 ± 21.0	NS
DIEPSS total score	3.1 ± 3.1	1.1 ± 1.7	NS
Total antipsychotics dose^a^	432 ± 112.6	342 ± 94.7	NS
Residence status (solitude/not solitude)	4/7	3/7	NS
Concomitant medication, n (%)			
Anticholinergic drugs	1 (9)	0 (0)	NS

*NS* no significant difference, *RLAI* risperidone long-acting injection, *PP* paliperidone palmitate, *PANSS* the positive and negative syndrome scale, *DIEPSS* the drug-induced extrapyramidal symptoms scale

^a^Chlorpromazine-equivalent dose

### Cognitive function

At baseline, the RLAI and PP groups did not differ in any of the BACS z-scores. In the analysis of the changes in the BACS z-scores from baseline to the study endpoint, the attention and processing speed score showed higher improvement in the PP group compared to the RLAI group (*p* = .039; partial eta-squared = .228; 95%CI = −.79 to -.02; Table 2). There were no significant intergroup differences in the changes in the other scores, including the total score of each scale.

**Table 2:** Change of primary outcome measures in risperidone long acting injection and paliperidone palmitate groups

	RLAI group				PP group				Difference in change between groups		
	Baseline		Change		Baseline		Change		*p*	Partial eta-squared	95%CI
	Mean	SD	Mean	SD	Mean	SD	Mean	SD			
BACS											
Verbal learning	6	1.04	.23	1.59	−1.34	.94	.06	1.22	.597	.017	−1.88 to 1.10
Working memory	−2.12	1.15	.12	.77	−1.10	.91	.26	.62	.412	.040	−1.09 to .470
Motor function	−2.65	.75	.28	.99	−2.74	1.09	.80	1.31	.374	.047	−1.80 to .71
Verbal fluency	−1.60	.83	.12	.50	−1.35	.97	.48	.60	.174	.106	−.93 to .18
Attention and processing speed	−2.65	1.37	.32	.36	−1.69	1.33	.62	.36	.039*	.228	−.79 to -.02
Executive function	−2.03	2.80	.63	2.69	−.44	1.09	−.13	.99	.171	.107	−1.76 to .34
Composite score	−2.22	.95	.29	.49	−1.44	.58	.35	.52	.290	.066	−.80 to .25

*p < .05

### Subjective well-being

No statistically significant differences were found for the all of baseline SWNS items score between the two groups. In the analysis of the changes in the SWNS scores from baseline to the endpoint, the emotional regulation score showed higher improvement in the PP group compared to the RLAI group (*p* = .034; partial eta-squared = .238; 95%CI = −5.90 to -.26; Table 3). There were no significant intergroup differences in the total score or in the changes in any of the other scores.

**Table 3:** Change of secondary outcome measures in risperidone long acting injection and paliperidone palmitate groups

	RLAI group				PP group				Difference in change between groups		
	Baseline		Change		Baseline		Change		*p*	Partial eta-squared	95%CI
	Mean	SD	Mean	SD	Mean	SD	Mean	SD			
SWNS											
Total score	79.09	19.93	−.09	19.58	67.00	19.25	3.20	7.36	.655	.012	−9.07 to 14.06
Mental function	16.00	4.52	−.18	4.58	12.60	3.92	.70	3.02	.990	< .001	−3.74 to 3.69
Self-control	15.45	4.44	1.46	5.18	14.20	3.49	.40	3.13	.267	.072	−1.91 to 6.48
Emotional regulation	16.55	4.06	−1.91	3.73	13.20	3.77	1.80	1.93	.034*	.238	−5.90 to -.26
Physical functioning	15.64	5.32	−1.27	5.95	13.80	4.54	.30	2.50	.825	.003	−4.89 to 3.951
Social integration	15.36	5.63	−.36	4.37	13.40	4.86	.00	2.16	.492	.028	−1.69 to 3.38
PANSS											
Total	83.00	19.88	−5.09	8.18	78.10	21.02	−1.70	5.08	.349	.049	−8.90 to 3.31
Positive	18.64	5.32	−2.00	2.65	16.30	4.72	−.30	2.00	.222	.082	−3.39 to .84
Negative	21.73	5.14	−.73	1.10	20.10	6.06	.20	1.62	.136	.119	−2.27 to .34
General psychopathology	42.64	10.88	−2.36	5.45	41.70	11.05	−1.60	2.50	.732	.007	−4.39 to 3.14
DIEPSS total	2.55	2.60	−.09	.30	0.90	1.28	.30	1.06	.220	.082	−1.23 to .30
Total antipsychotic dose^a^	432	112.6	−12.6	18.6	342	94.7	−26.8	52.0	.651	.012	−30.41 to 47.43

^a^Chlorpromazine-equivalent dose, *p < .05

### Changes in PANSS scores, DIEPSS total scores and dose of antipsychotic drugs

There were no significant differences in PANSS score, DIEPSS total score, or antipsychotic doses at baseline between the two groups. In addition, the changes in these scores from the baseline to endpoint did not differ between the two groups (Table 3).

### Associations between changes in BACS scores and changes in SWNS scores

The changes in the SWNS total score did not correlate with the changes in any of the BACS z-scores. However, a correlation was found between improvements in the BACS score of attention and processing speed and the SWNS subscale of emotional regulation (*r* = .46, *p* = .037; Table 4). There were no significant correlations between any of the other BACS scores and any of the other subscales of the SWNS.

**Table 4:** Correlations between changes in BACS scores and changes in SWNS scores

		BACS						
		VL	WM	MoF	VF	AP	EF	COM
SWNS	TS	−.03	−.05	.03	−.24	.29	.17	.08
	MeF	−.07	.13	.05	−.19	.24	.33	.21
	SC	−.28	.01	.02	−.27	.06	.16	−.08
	ER	.11	−.15	.23	.09	.46*	−.03	.20
	PF	−.13	.03	.14	−.20	.09	.26	.14
	SI	.08	−.05	.03	-.13	.23	.33	.24

*BACS* brief assessment of cognition in schizophrenia, *SWNS* subjective well-being under neuroleptic drug treatment short form, *VL* verbal learning, *WM* working memory, *MF* motor function, *VF* verbal fluency, *AP* attention and processing speed, *EF* executive function, *COM* composite score, *TS* total score, *MF* mental function, *SC* self-control, *ER* emotional regulation, *PF* physical functioning, *SI* social integration

*p < .05

## Discussion

To the best of our knowledge, the present randomized study is the first attempt to evaluate changes in cognitive function in patients with schizophrenia during RLAI or PP treatment. The most clinically relevant finding obtained by this preliminary study is that patients who switch from RLAI to PP might have higher improvement in attention and processing speed than those who continue treatment with RLAL. Although the differences in effects on cognitive function observed between the two groups were very small, our results might have some implications for the treatment of schizophrenia.

To date, a number of studies have investigated the influence of pharmacological therapy on cognitive function in patients with schizophrenia. Many reports have demonstrated a slightly to moderately favorable influence of antipsychotic treatment on the cognitive function of patients with schizophrenia [30]. However, some studies have reported that cognitive function is negatively affected by standard- and high-dose antipsychotic treatment [31–34], antipsychotic polypharmacy [27], concomitant use of anti-Parkinson’s disease drugs [35], or concomitant use of benzodiazepines [36]. Thus, particular attention to drug doses and combinations is the first measure to avoid cognitive dysfunction. As a further step, the effects of different antipsychotics on cognitive function have been examined by several studies. Differences between the impact of SGAs and first-generation antipsychotics (FGAs) on cognition have frequently been reported [37]. However, studies focused on the possible different effects of different SGAs found both negative [38, 39] and positive results [13, 40, 41]. Given that individual SGAs show different pharmacological profiles and that cognitive function consists of diverse domains, it seems likely that the effects on cognitive function may differ (even if slightly) among drugs [42].

Regarding the present study, the different pharmacokinetic and pharmacodynamic properties between RLAIs and PPs should be considered. The greatest pharmacokinetic difference between the two compounds pertains to the PAL/RIS drug level ratio in the blood. PP works exactly as PAL, while the PAL/RIS blood ratio during RLAI treatment (both 25, 37.5, and 50 mg/14 days) was reported to be 2.4–3.0 [43] and much higher during Oral-RIS treatment (2 mg/day: 13.3, 4 mg/day: 11.5, 6 mg/day: 13.3). One possible reason for such a low PAL/RIS for RLAI is the lack of a first pass effect in the liver. This suggests that RLAI is a drug that more strongly reflects the influence of RIS compared to PP and Oral-RIS.

Under the pharmacodynamic point of view, PAL and RIS show many similarities in their affinities for receptors. However, their affinities for the alpha 2-adrenergic receptor are different, since PAL has a higher affinity than RIS for this receptor [12]. Animal studies have shown that the blockage of alpha 2-adrenergic receptors greatly affects the release of dopamine and noradrenaline from the medial prefrontal cortex [44]. Clinical studies have demonstrated that treatment with antidepressants with strong alpha 2-adrenergic receptor antagonistic activity (e.g. mirtazapine [45] or mianserin [46]) may improve cognitive function. Furthermore, many studies in humans, monkeys, and rodents have demonstrated that the noradrenergic nervous system, particularly through the alpha 2-adrenergic receptor, contributes to the regulation of attention [47]. Given that attention is a markedly disturbed cognitive domain in schizophrenia, the results of the present study deserve to be investigated by future studies. The results of a previous study [13] that compared Oral-RIS and Oral-PAL are poorly comparable with the present ones, since the reported pharmacokinetic differences among RLAIs, PPs, Oral-RIS, and Oral-PAL. In addition to these differences, the following issues should be considered: (1) the range of fluctuations in blood levels of Oral-RIS and Oral-PAL differ substantially (peak-to-trough fluctuation: 1.47 for Oral-PAL, 3.30 for Oral-RIS) [48]; (2) the frequency of sedation is higher with Oral-RIS than with Oral-PAL [49]; and (3) Oral-RIS may adversely affect working memory, a cognitive functional domain related to short-term memory [50].

In the present study, there were no differences between the two groups in the change in the total scores of the PANSS and DIEPSS. These results suggest similar efficacy and tolerability of RLAI and PP, consistently with previous studies [51–53].

The total score of the SWNS did not differ between the two groups, and there were no correlations between the changes in the total SWNS score and the BACS items. Thus, the subjective well-being of patients with schizophrenia did not differ between RLAI and PP, despite the higher improvement in some cognitive domains observed in the PP group. However, a correlation between emotional regulation (a SWNS subscale) and attention/processing speed improvement during treatment was found. Gross et al. assumed a model of emotion regulation that includes five phases and hypothesized that attention plays an important role in this process [54]. The event-related potentials measured by an electroencephalographic study and a functional magnetic resonance imaging study demonstrated a relationship between attention and emotional regulation [55, 56]. In addition, numerous reports have examined the dysfunctional interactions between attention and unpleasant stimuli, which are particularly interesting in regard to schizophrenia [57]. However, the current evidence appears still insufficient to state that improvements in attention during the pharmacological treatment of schizophrenia can result in improvements of emotional regulation [57]. In addition, the SWNS subscales may be greatly affected by the total score and they may have low reliability [58]. Thus, further studies focused on this issue are needed.

This study has several limitations. First, the small sample size increases the risk of false negative findings. The lack of multiple-testing correction (e.g. Bonferroni correction) may also result in type I errors, but given the pilot nature of the present study, the results should be considered preliminary. Therefore, replication studies are needed, possibly with larger samples and through a randomized, double blind design. Second, the open-label design might have affected the results, since the expectations of patients or raters might have affected the assessments. However, the similar effects of the two drugs on symptomatology scores do not support this bias and the randomized design represents a point of strength. Third, we did not consider the drug blood level at the time of the cognitive assessment or of the other assessments. The drug blood level may have affected these evaluations [31], despite drug level fluctuations for LAIs are considerably smaller than for oral antipsychotics [59]. Finally, we did not strictly limit the use of anticholinergic agents, which may have an impact on cognitive function. However, only one patient in the RLAI group used anticholinergic agents, with probably no impact on the present results. Future studies should fix the timing of cognitive assessments and strictly control the use of concomitant medications.

## Conclusions

To the best of our knowledge, this is the first study to compare the influence of RLAI and PP on cognitive functional domains and well-being. Compared to continue treatment with RLAI, switching from RLAL to PP therapy may have more favorable effects on attention and processing speed (as assessed using the BACS scale), which are important elements in cognitive function, although there were neither differences in symptom improvement according to the PANSS, nor in the frequency of extrapyramidal side effects. The higher improvement in attention and processing speed found in the PP group was associated with higher improvement in emotional regulation (accordingly to the SWNS). However, these results are preliminary and independent replication in larger samples is required before any definitive statement.

## Abbreviations

BACS, brief assessment of cognition in schizophrenia; DIEPSS, drug-induced extrapyramidal symptoms scale; FGAs, first-generation antipsychotics; JART, japanese adult reading test; LAI, long-acting injection; PAL, paliperidone; PANSS, positive and negative syndrome scale; PP, paliperidone palmitate; RIS, risperidone; RLAI, risperidone long-acting injection; SGAs, second-generation antipsychotics; SWNS, subjective well-being under neuroleptic drug treatment short form

## Additional file

Additional file 1: CONSORT 2010 checklist of information to include when reporting a randomised trial. (PDF 136 kb)
